# SCM-198 Alleviates Endometriosis by Suppressing Estrogen-ERα mediated Differentiation and Function of CD4^+^CD25^+^ Regulatory T Cells

**DOI:** 10.7150/ijbs.68224

**Published:** 2022-02-21

**Authors:** Yun-yun Li, Yi-kong Lin, Yue Li, Xin-hua Liu, Da-jin Li, Xiao-lin Wang, Li Wang, Yi-zhun Zhu, Min Yu, Mei-rong Du

**Affiliations:** 1 NHC Key Lab of Reproduction Regulation (Shanghai Institute of Planned Parenthood Research), Shanghai Key Laboratory of Female Reproductive Endocrine Related Diseases, Hospital of Obstetrics and Gynecology, Fudan University Shanghai Medical College, Shanghai, China.; 2 State Key Laboratory of Quality Research in Chinese Medicine and School of Pharmacy, Macau University of Science and Technology, Macau SAR, China.; 3 Shanghai Key Laboratory of Bioactive Small Molecules, Department of Pharmacology, School of Pharmacy, Fudan University, Shanghai, China.; 4 Shanghai JIAI Genetics & IVF Institute, Obstetrics & Gynecology Hospital of Fudan University, Shanghai, China.; 5 Department of Obstetrics and Gynecology, Guangzhou First People's Hospital, School of Medicine, South China University of Technology, Guangzhou, China.

**Keywords:** SCM-198, EMS, Tregs, estrogen, ERα

## Abstract

**Background:** Endometriosis (EMS), a typical endocrine immune disorder, associates with dramatically increased estrogen production and disorganized immune response in ectopic focus. Peritoneal regulatory T cells (Tregs) expansion in women with EMS and their pathogenic role attributable to endometriotic immunotolerance has been reported. Whether local high estrogen promotes EMS by discipling Tregs needs to be further explored. Up to date, there is no effective medicine for the treatment of EMS. SCM-198 is a synthetic leonurine with multiple physiological activities. Whether SCM-198 could regulate Tregs via estrogen and facilitate the radical cure of EMS has not yet been reported.

**Methods:** Proportion of Tregs in peritoneal fluid of patients with EMS was firstly analyzed via flow cytometry. Peritoneal estrogen concentration and the mRNA levels of estrogen receptor α (ERα) and estrogen receptor β (ERβ) of Tregs were detected by ELISA and RT-PCR, respectively. Grouped *in vitro* induction assays were performed to explore the effects of SCM-198 and estrogen signaling on Tregs. Cell invasion and viability assays were utilized to detect the crosstalk between Tregs and ectopic endometrial stromal cells (eESCs), with or without SCM-198 treatment. Furthermore, EMS mice models were established to verify the therapeutic effects of SCM-198.

**Results:** Increased Tregs were found in peritoneal fluid of EMS patients, accompanied with estrogen-ERα overactivation. Estrogen-ERα triggered the expansion of Tregs and their cytokine production (IL-10 and TGF-β1), which could be reversed by SCM-198 treatment. Moreover, SCM-198 abated the invasion and viability of eESCs enhanced by Tregs. *In vivo* experiments confirmed that SCM-198 obviously retarded the growth of ectopic lesions and downregulated the functions of Tregs via estrogen-ERα inactivation.

**Conclusions:** These data suggest that SCM-198 attenuates Tregs expansion via the inhibition of estrogen-ERα signaling in EMS and offer a promising therapy for such a refractory disease.

## Introduction

Endometriosis (EMS), a refractory estrogen-dependent disease, is defined as the growth of endometrial tissue outside the uterine cavity 1, 2. As a common gynecological disease, the prevalence of EMS dramatically reaches 10% in 2020 with 50% recurrent rate 3-5. Ectopic endometrial tissue growth and its periodic bleeding leads to inflammation and fibrosis, which eventually result in chronic pelvic pain and dysmenorrhea. The long-term presence of EMS give rise to estimated 30%-50% incurable infertility, which deserving more attention in patients of reproductive age 6, 7. Although, extensive research has been carried out to verify the role of estrogen dependence and progesterone resistance in EMS, mechanisms require further deepening to elucidate the occurrence and development of EMS. Furthermore, a new therapeutic strategy is urgently needed to alleviate suffering and tackle such an incurable disease.

Actually, immune disorder cannot shirk the responsibility in the progress of EMS 8, 9. Weakened phagocytosis ability of macrophages and reduced cytotoxicity of natural killer (NK) cells were found in peritoneal fluid of EMS, which were tightly linked with ectopic lesion formation 10-12. Regulatory T cells (Tregs) have been extensively studied in the region of oncology for their important and irreplaceable role on immune tolerance 13, 14. Based on the tumor-like immune suppressive microenvironment, an emerging focus in the pathogenesis of EMS has been put on the role of Tregs. Several studies have pointed out Tregs are more abundant in peritoneal fluid of women with EMS 15-17, implying the important role of Tregs on the development of EMS. Moreover, higher production of IL-10 and TGF-β1 in Tregs potentiate ectopic lesions' attachment, survival and growth 18. Published researches are biased towards the changes in the proportion of peritoneal Tregs in EMS, while few studies have explored the reasons for the accumulation of Tregs and their influence on the growth of EMS. Meanwhile, whether Tregs could consider as a therapeutic target of EMS remains unknown.

*Leonurus japonicus* Houtt has long been used to treat obstetric and gynecological diseases, such as irregular menstruation, dysmenorrhea. SCM-198 is a synthetic form of leonurine, which is an active alkali component purified from *Leonurus japonicus* Houtt 19, 20. Our previous study found that SCM-198 relieved the oxidative stress of endometrial stromal cells (ESCs) 21 and had a therapeutic effect on LPS-induced endometritis 22. Moreover, Liu et al found that leonurine attenuated the hyperalgesia in mice with induced adenomyosis 23. However, study focused on the therapeutic effects of SCM-198 on EMS has not been reported.

Herein, we focused on the regulatory impact of SCM-198 on peritoneal Tregs and estrogen signaling. We found that SCM-198 remarkably attenuated estrogen secretion of ectopic lesions and abated estrogen receptor α (ERα) activation of Tregs. Then, we observed a dramatical reduction of expansion in peritoneal Tregs induced by estrogen-ERα after SCM-198 treatment. More important, we demonstrated that SCM-198 administration effectively lessened the ectopic lesions in EMS mice models, with a significant decrease of peritoneal Tregs. Accordingly, our data suggest that SCM-198 might be a promising therapy for EMS.

## Materials and Methods

### Human sample collections

We recruited 76 women undergoing laparoscopic surgery for ovarian endometriosis, uterine leiomyoma or infertility, etc., at Obstetrics and Gynecology Hospital of Fudan University. Inclusion criteria for EMS utilized in this study were patients diagnosed with EMS by laparoscopy or histology without the combination of adenomyosis or uterine leiomyoma or polycystic ovary syndrome (PCOS) (age < 48 years). Inclusion criteria for non-EMS were patients diagnosed with non-EMS diseases, such as adenomyosis, uterine leiomyoma, benign teratoma or infertility (age < 48 years). Patients who had received medical/hormonal supplementation during the last 3 months or diagnosed with malignant tumor or pelvic inflammatory disease were excluded from both EMS and non-EMS patients' groups. Among them, 46 EMS peritoneal fluid (ePF) and 30 non-EMS peritoneal fluid (nPF) were collected. Ectopic endometrial tissues were obtained from EMS patients (aged 22-45 years) via laparoscopy. The collection and use of these samples were approved by the Human Research Ethics Committee of the Obstetrics and Gynecology Hospital of Fudan University (No. Kyy2016-4) (Shanghai, China).

### Isolation and culture of human ectopic endometrial stromal cells

Briefly, the ectopic endometrial tissues were minced (2-3-mm pieces) and digested in DMEM/F-12 containing collagenase type IV (0.1%, Sigma, USA) for 30 min at 37 °C. The dispersed cells were then filtered through a 400-mesh wire sieve to remove the undigested tissue pieces containing the glandular epithelium. After gentle centrifugation, the supernatant was discarded, and the cells were resuspended in DMEM/F-12 containing 10% fetal bovine serum (FBS) (Gemini, USA), 100 IU/ml penicillin (Sigma, USA), 100 μg/ml streptomycin (Sigma, USA), and 1 μg/ml amphotericin B (Sangon, China) at 37 °C in 5% CO_2_.

### Flow Cytometry

Cell surface and intracellular molecular expressions were evaluated by flow cytometry using CytoFLEX (Beckman Coulter, USA). Fluorescein-conjugated anti-mouse antibodies, including CD3-BV785/BV510/AF700, CD4-BV510/BV605, CD25-FITC, FOXP3-PE, IL-10-APC, TGF-β1-BV421 (Biolegend, UK), and anti-human antibodies, including CD3-BV510, CD4-BV650, CD25-APC, FOXP3-PE, IL-10-BV421, TGF-β1-PE/CY7 (Biolegend, UK), were used according to the manufacturer's procedures. For cell-surface staining, single-cell suspensions were stained on ice for 30min in PBS with 1% FBS. For intracellular staining, cells were fixed and permeabilized using the Fix/Perm kit (Biolegend, UK). To detect intracellular cytokines, cells were stimulated for 4 h with Cell Activation Cocktail (with Brefeldin A) (Biolegend, UK). Thereafter, cells were harvested, stained for surface expression, and then fixed and permeabilized for intracellular staining. Flow cytometry data were analyzed using FlowJo software (BD, UK).

### ELISA

Collect cell supernatant of nPF, ePF and ectopic endometrial stromal cells (eESCs) to evaluate the levels of estrogen. The concentration of estrogen was assayed by ELISA according to the manufacturer's instructions (estrogen, #CSB-E07286h, China).

### Quantitative real-time PCR

Total RNA was extracted using TRIzol reagent (Invitrogen, USA) and then reverse-transcribed into first-strand complementary DNA (cDNA) (Takara, Japan) by using PrimeScript RT Master Mix (#RR036A, TaKaRa, Japan) according to the manufacturer's instructions. The synthesized cDNA was amplified with specific primers and SYBR Green (Takara, Japan) using an ABI PRISM 7900 Sequence Detection System (Applied Biosystems, USA). Triplicate samples were examined for each condition. A comparative threshold cycle value was normalized for each sample using the *2*^-ΔΔ^Ct method.

### Isolation of CD4^+^CD25^-^ T cells and CD4^+^CD25^+^ Tregs

Peripheral blood mononuclear cells (PBMCs) isolated from healthy women were separated by use of lymphoprep (#07851, STEMCELL, Canada) based on gradient centrifugation. CD4^+^ T cells are obtained by negative selection, and CD25^+^ cells are obtained by positive selection according to the magnetic beads sorting instructions (#130-091-301, Miltenyi, Germany).

### *In vitro* generation of CD4^+^CD25^+^ Tregs

CD4^+^CD25^+^ Tregs were generated as previously described 24. Coat a 24-well plate with 10 µg/ml of anti-CD3 mAb (Biolegend, UK) at 37 °C for 2 h and then wash the plate once with PBS before cell plating. Plate 1 × 10^6^ CD4^+^CD25^-^ T cells into the precoated well and add anti-CD28 mAb (Biolegend, UK) (2 µg/ml), TGF-β1 (5 ng/ml, PeproTech, USA) and rhIL-2 (20 ng/ml, Biolegend, UK) for 72h incubation. At 24th hour induction, add 17β-estradiol (E2) (#E2758, Sigma, USA) (100 nM), ERα-specific agonist propyl pyrazole triol (PPT) (#HY-100689, MCE, USA) (5 nM), SCM-198 (200 µM), E2+SCM-198 (100 nM, 200 µM) or PPT+SCM-198 (5 nM, 200 µM) to the cells for the rest 48h.

### Cell invasion assay

CD4^+^CD25^+^ Tregs were collected and washed with PBS for three times after the pretreatment with E2 (100 nM), E2+SCM-198 (100 nM, 200 µM), PPT (5 nM), or PPT+SCM-198 (5 nM, 200 µM) for 48 h. Then, eESCs were cocultured with the pretreated Tregs or SCM-198 (0, 50, 100, 200 µM) for 48h.

Spread 20 µl matrigel (BD, USA) on the upper layer of the chamber (Corning, USA) and then culture it at 37 °C for 2 h. Add 5× 10^4^ eESCs (200 µl) to the upper chamber, which containing 10% FBS. Add 800 µl medium, which containing 20% FBS, to the lower chamber. Incubate cells at 37 °C for 48 h. The cells were fixed with 4% formaldehyde solution for 30min, and then stained with crystal violet (Beyotime, China) for 30 min. Observe the invasion with upright microscope (Olympus, Japan).

### Cell viability assay

eESCs were cocultured with SCM-198 (0, 50, 100, 200, 400 µM) or CD4^+^CD25^+^ Tregs, which were washed with PBS for three times after the pretreatment with E2 (100 nM), E2+SCM-198 (100 nM, 200 µM), PPT (5 nM), or PPT+SCM-198 (5 nM, 200 µM) for 48 h.

According to the instructions, add 10 µl CCK8 detection reagent (Dojindo, Japan) and 90 µl fresh culture medium to each well. Measure the absorbance at 450 nm wavelength to detect cell viability.

### Mouse EMS model

Female C57BL/6 mice (6 weeks old) were purchased from Shanghai JieSiJie Laboratory Animal Co., Ltd. SCM-198 was chemically synthesized by Dr Zhu Yizhun's lab. Permission for animal experimental was provided by the Research Ethics Committee of Obstetrics and Gynecology Hospital of Fudan University.

After two weeks adaptation, mice were randomly selected as the donors of EMS models. Donor mice were intraperitoneally injected with E2 (0.2 µg/g bodyweight) three times for one week. Vaginal smears were utilized to select estrus mice as the recipients of EMS mice models. Mixture of the donor mouse uterine fragments and PBS were intraperitoneally injected to recipient mouse. Recipient mice were allowed to rest for one week.

To investigate the effects of SCM-198 on EMS, recipient mice were randomly divided into three groups: EMS group, EMS + low-dose SCM-198 group (EMS+SCM-198 L, 7.5 mg/kg) and EMS + high-dose SCM-198 group (EMS+SCM-198 H, 15 mg/kg). Equal number mice were taken as control group. To investigate the effects of SCM-198 on estrogen treated EMS, recipient mice were randomly divided into four groups: EMS group, EMS + E2 group (150 µg/kg), EMS + SCM-198 group (15 mg/kg), EMS + E2 + SCM-198 group (150 µg/kg, 15 mg/kg). Intraperitoneally inject 200 µl of E2 or SCM-198 to the recipient according to the corresponding dose (once a day for total one week). EMS group were given the same dose and frequency of PBS and the control group received no treatment. One week later, the mice were sacrificed. Collect EMS ectopic tissues, uteri and peritoneal fluid for subsequent treatment.

### Western blotting analysis

The total proteins of ectopic lesions in EMS mice were extracted using radio-immunoprecipitation assay (RIPA) buffer (Beyotime, China) supplemented with protease and phosphatase inhibitors (Sigma, USA). The protein concentration was measured using a BCA protein assay kit (Beyotime, China). After denaturation, equal amounts of proteins were separated via SDS-polyacrylamide gel electrophoresis (PAGE) before wet transfer onto polyvinylidene difluoride membranes. Nonspecific binding sites were blocked by incubating the membranes with 5% skim milk in Tris-buffered saline with 0.1% Tween 20 (TBS-T) for 1 h. Then, the membranes were incubated overnight at 4 °C with primary antibodies (1:1000) against MMP9 (#ab228402, Abcam, UK), MMP2 (#87809, CST, USA), BCL2 (#2870, CST, USA) and GAPDH (#10112, Arigo, China). Subsequently, membranes were incubated with appropriate horseradish peroxidase-conjugated anti-rabbit (#65351, Arigo, China) or anti-mouse (#65350, Arigo, China) IgG secondary antibodies for 1h at room temperature. After three washes with TBS-T, immunopositive bands on the blots were visualized using chemiluminescent HRP substrate (#WBKLS0100, Millipore, USA) on the enhanced chemiluminescence detection system (Merck Millipore, USA).

### Statistical analysis

Flow cytometry data were analyzed using FlowJo software (BD, UK). Prism 8 software (GraphPad, USA) was used for data analysis. Statistical significance was determined using Student's t-test for 2-group or one-way ANOVA for multiple group comparisons. The data are presented as mean±SEM. Statistical significance was attained when P < 0.05.

## Results

### Tregs accumulates in peritoneal fluid of EMS patients

**Figure 1:**
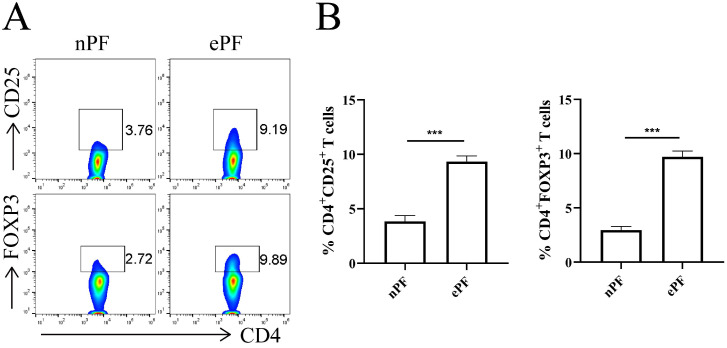
** Tregs were accumulated in peritoneal fluid of EMS patients. (A)** Representative pictures showing the analysis of CD25 and FOXP3 expression in CD4+T cells of nPF and ePF. **(B)** Quantitative flow cytometry results for CD25 and FOXP3 expression in CD4+T cells of nPF and ePF (n=10). Data are presented as the mean ± SEM. ***P < 0.001.

The demographic and obstetrical characteristics of enrolled participants were summarized in Table 1. Flow cytometry experiments were conducted to evaluate the change of peritoneal Tregs in EMS. Tregs were labeled with surface marker CD25 and transcription factor FOXP3. The results showed that approximately 3.8% of CD4^+^T cells were CD25 positive and 3.0% of CD4^+^T cells were FOXP3 positive in nPF (Fig. 1A and 1B). Whereas, the percentage of CD25^+^ and FOXP3^+^ in CD4^+^T cells increased to 9.3% and 9.7% in ePF (Fig. 1A and 1B). Compared with control groups, peritoneal Tregs remarkably increased in EMS, suggesting the possible roles of Tregs in the development of EMS.

### Estrogen-ERα signaling potentiates the expansion of Tregs

**Figure 2:**
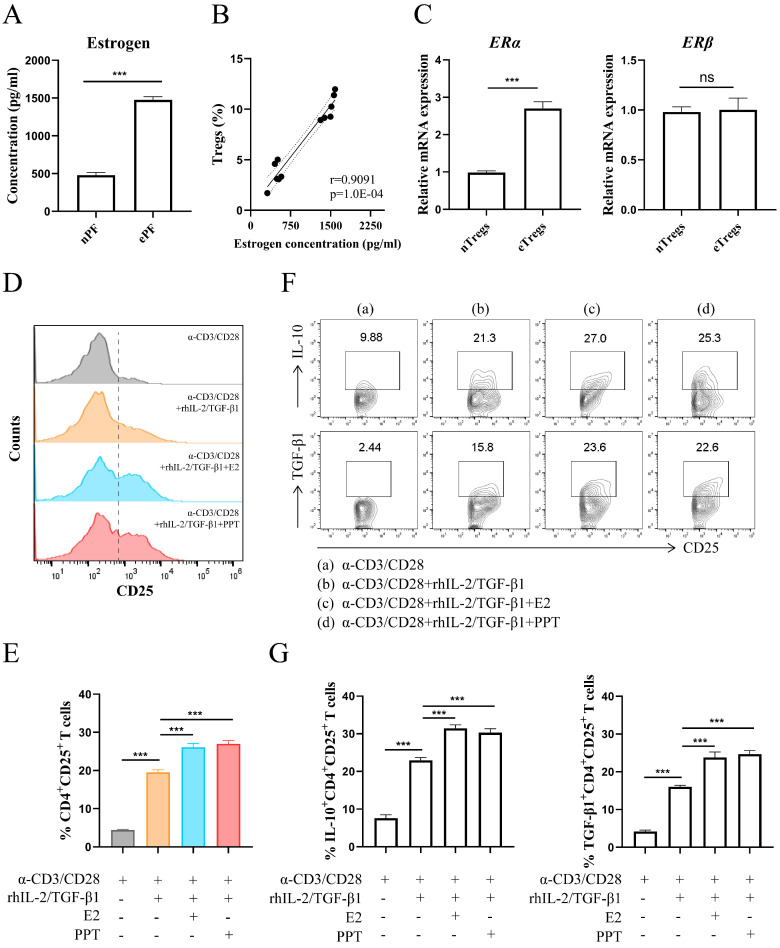
** Estrogen-ERα signaling potentiated the expansion of Tregs. (A)** The concentrations of estrogen in nPF and ePF were measured by ELISA (n=6). **(B)** Spearman correlation of estrogen concentration and Tregs' proportion. **(C)** The mRNA expression of ERα and ERβ in CD4^+^CD25^+^ Tregs from nPF and ePF (n=5). **(D-G)** CD4^+^ CD25^-^ T cells from PBMCs of healthy women were cultured in the presence of anti-CD3 (10 µg/ml) mAb+ anti-CD28 (2 µg/ml) mAb + TGF-β1 (5 ng/ml) + rhIL-2 (20 ng/ml) for total 72 h and at the 24^th^ hour, treated with or without E2 (100 nM) or PPT (5 nM). The cells were harvested and evaluated by flow cytometry for CD25 expression in CD4^+^T cells (D, E), and for IL-10, TGF-β1 expression in CD4^+^CD25^+^ Tregs (F, G) (n=9). Data are presented as the mean ± SEM. ***P < 0.001; ns, not significant.

The results in Fig. 2A showed a predominance of estrogen in EMS via ELISA assay. There was a highly positive correlation between the concentration of estrogen and the proportion of Tregs (Fig. 2B). The transcriptional levels of estrogen receptors in Tregs were assessed via RT-PCR and higher ERα mRNA level was observed in Tregs from ePF (Fig. 2C). While no significant change of estrogen receptor β (ERβ) mRNA was detected in Tregs (Fig. 2C). Therefore, we choose ERα as the follow-up research object. To assess whether there is a correlation between Tregs and estrogen-ERα signaling, CD4^+^CD25^-^ T cells purified from PBMCs were cultured with α-CD3, α-CD28, rhIL-2 and TGF-β1 to mimic the process of Tregs differentiation *in vitro*, with or without E2 or PPT. The results revealed that E2 and PPT both promoted expansion of Tregs (Fig. 2D and 2E). In addition, E2 and PPT notably increased the production of IL-10 and TGF-β1 in Tregs (Fig. 2F and 2G).

Collectively, these data indicate that estrogen-ERα signaling promotes the differentiation of Tregs and their production of immunosuppressive cytokines (IL-10 and TGF-β1).

### SCM-198 abates the differentiation and cytokine production of Tregs by inhibiting estrogen-ERα signaling

**Figure 3:**
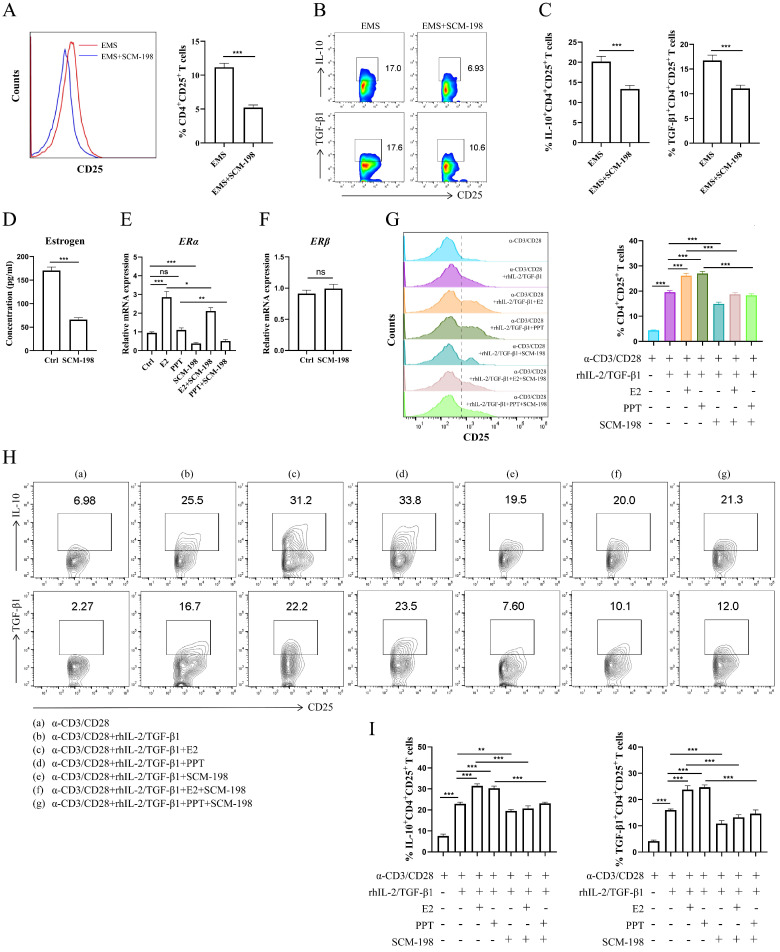
** SCM-198 abated the differentiation and cytokine production of Tregs by inhibiting estrogen-ERα signaling. (A-C)** The total peritoneal immune cells from ePF were cultured with SCM-198 (200 µM) for 48 h. The proportions of CD4^+^CD25^+^ Tregs were detected by flow cytometry (A) (n=10). The expression of IL-10 and TGF-β1 in CD4^+^CD25^+^ Tregs were analyzed by flow cytometry (B, C) (n=10). **(D)** eESCs were treated with SCM-198 (200 µM) for 48h. The concentration of estrogen produced by eESCs were measured by ELISA (n=4). **(E)** The mRNA expression of ERα in Tregs treated with or without E2 (100 nM), PPT (5 nM), SCM-198 (200 µM), E2+SCM-198 (100 nM, 200 µM) or PPT+SCM-198 (5 nM, 200 µM) for 48 h were measured by RT-PCR (n=5). **(F)** The mRNA expression of ERβ in Tregs treated with or without SCM-198 (200 µM) for 48 h were measured by RT-PCR (n=5). **(G-I)** CD4^+^ CD25^-^ T cells from PBMCs were cultured in the presence of anti-CD3 (10 µg/ml) mAb + anti-CD28 (2 µg/ml) mAb + TGF-β1 (5 ng/ml) + rhIL-2 (20 ng/ml) for total 72h and at the 24th hour, treated with or without E2 (100 nM), PPT (5 nM), SCM-198 (200 µM), E2+SCM-198 (100 nM, 200 µM) or PPT+SCM-198 (5 nM, 200 µM). The cells were harvested and evaluated by flow cytometry for CD25 expression in CD4^+^T cells (G), and for IL-10, TGF-β1 expression in CD4^+^CD25^+^ Tregs (H, I) (n=9). The Data are presented as the mean ± SEM. *P < 0.05, **P < 0.01, ***P < 0.001; ns, not significant.

To explore the regulatory effects of SCM-198 on Tregs, we cocultured the total peritoneal immune cells from ePF with SCM-198. The data showed that SCM-198 obviously downregulated the proportion of Tregs (Fig. 3A). Lowered IL-10 and TGF-β1 levels of Tregs were also detected after SCM-198 treatment (Fig. 3B and 3C). To assess whether SCM-198 regulated estrogen production, we treated eESCs, the main source of estrogen in ePF, with SCM-198. The results revealed that SCM-198 notably suppressed estrogen production in eESCs (Fig. 3D). Moreover, SCM-198 suppressed the mRNA level of ERα in Tregs (Fig. 3E). While, no significant change was detected in ERβ with SCM-198 treatment (Fig. 3F). To confirm whether SCM-198 inhibited Tregs by suppressing estrogen-ERα signaling, we conducted *in vitro* differentiation assay of Tregs under the treatment of SCM-198. As shown in Fig. 3G, SCM-198 dramatically decreased the expansion of Tregs induced by E2 or PPT. Furthermore, the production of IL-10 and TGF-β1, which were reinforced by E2 or PPT, can also be reversed by SCM-198 (Fig. 3H and 3I). These findings demonstrate that SCM-198 can suppress Tregs accumulation and their immunosuppressive cytokines production via estrogen-ERα signaling inhibition.

### SCM-198 ameliorates the invasion and viability of eESCs enhanced by Tregs

**Figure 4:**
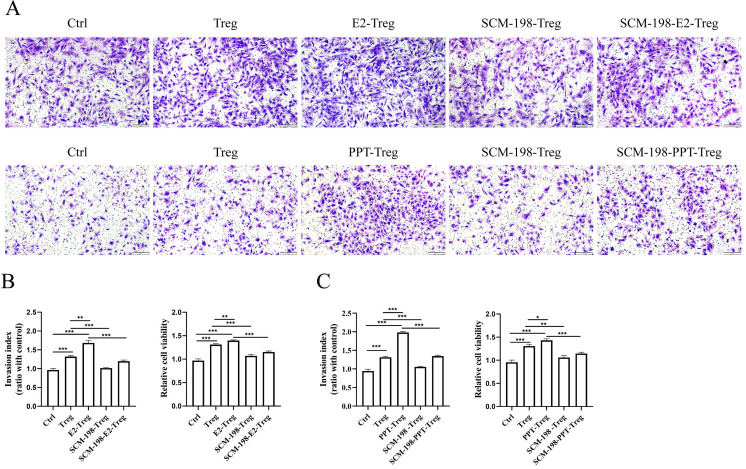
** SCM-198 ameliorated the invasion and viability of eESCs enhanced by Tregs.** CD4^+^ CD25^+^Tregs from ePF were cultured with or without E2 (100 nM), E2+SCM-198 (100 nM, 200 µM), PPT (5 nM), or PPT+SCM-198 (5 nM, 200 µM) for 48 h. Then, the pretreated Tregs were cocultured with eESCs for another 48 h. **(A)** Representative pictures for the invasion ability of eESCs treated as indicated. Scale bar, 200 µm. **(B, C)** Data depicted the invasion index and cell viability of eESCs treated as indicated. The invasion index of eESCs was normalized to that of the control (n=4). The viability of eESCs was measured by CCK8 and cell viability was normalized to that of the control (n=6). The Data are presented as the mean ± SEM. *P < 0.05, **P < 0.01, ***P < 0.001.

The occurrence and development of EMS mainly depends on the augmented invasion and viability of eESCs. We first explored the direct effects of SCM-198 on the biological behavior of eESCs. The results revealed that SCM-198 had little effect on the invasion, but could restrain the growth of eESCs (Fig. S1A-S1C). Studies have shown that Tregs could stimulate the invasion and viability of eESCs 18. Therefore, we further explored whether SCM-198 could attenuate the abnormal interaction between Tregs and eESCs. The results showed that Tregs facilitated the invasion and viability of eESCs, which could be enhanced by the pretreatment of E2 and PPT (Fig. 4A-4C). Reversely, SCM-198 significantly diminished the promotive effects induced by E2 or PPT (Fig. 4A-4C). These results suggest that SCM-198 alleviates the promotion of Tregs on eESCs' invasiveness and growth induced by estrogen-ERα signaling.

### SCM-198 suppresses the growth of EMS and downregulates the accumulation of Tregs in EMS mice

**Figure 5:**
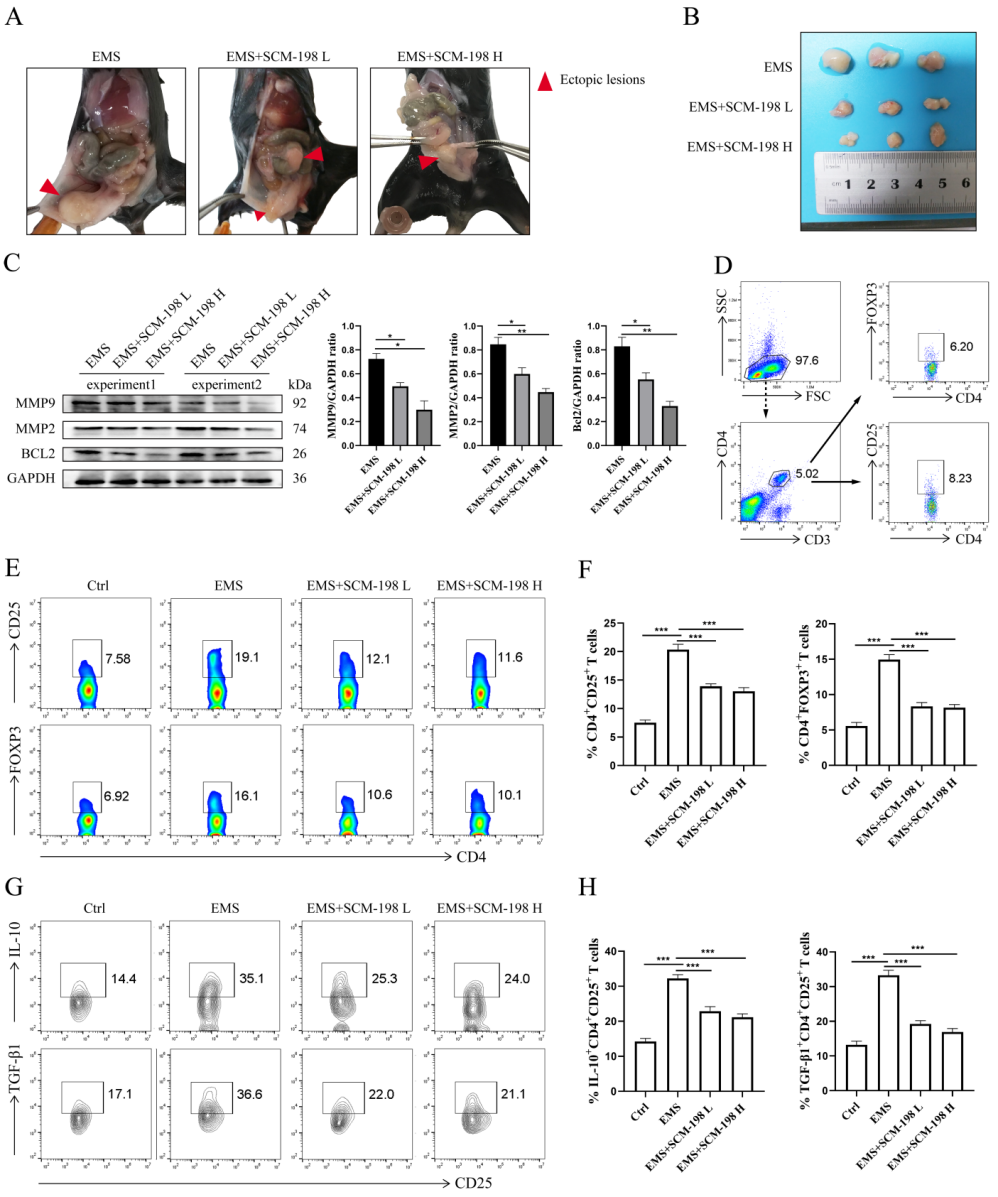
** SCM-198 suppressed the growth of EMS and downregulated the accumulation of Tregs in EMS mice. (A)** Representative images of the overall condition of ectopic lesions in the abdominal cavity of EMS mice models treated with or without SCM-198 at low (EMS+SCM-198 L, 7.5 mg/kg) or high (EMS+SCM-198 H, 15 mg/kg) dose once a day for one week. **(B)** Representative image of ectopic lesions from EMS mice under SCM-198 administration. **(C)** The protein levels of MMP9, MMP2 and BCL2 of ectopic lesions were detected by western blotting (n=4). **(D)** Representative images showing the gating strategy for Tregs in peritoneal fluid of EMS mouse. **(E, F)** Representative (E) and quantitative (F) flow cytometry results for CD25 and FOXP3 expression in CD4^+^T cells from peritoneal fluid of control and EMS mice (n=10). **(G, H)** Representative (G) and quantitative (H) results showing the analysis of IL-10 and TGF-β1 expression in CD4^+^ CD25^+^ Tregs from control and EMS mice (n=10). Data are presented as the mean ± SEM. *P < 0.05, **P < 0.01, ***P < 0.001.

Next, we investigated the therapeutic effects of SCM-198 on *in vivo* EMS mice models. Fig. 5A exhibited the overall condition of ectopic lesions in the abdominal cavity of EMS mice models under SCM-198 administration. As shown in Fig. 5B, SCM-198 significantly suppressed the growth of ectopic lesions at both low and high dose. SCM-198 appreciably constrained the invasion and viability of ectopic lesions by downregulating the protein levels of MMP9, MMP2 and BCL2 (Fig. 5C). Consistent with the above results observed in human ePF, the proportion of Tregs was obviously upregulated in mice ePF, compared with that from nPF (Fig. 5D-5F). The production levels of IL-10 and TGF-β1 in Tregs were also increased in EMS groups (Fig. 5G and 5H). Similarly, SCM-198 administration significantly reduced the proportion of Tregs (Fig. 5E and 5F) and decreased the production of IL-10 and TGF-β1 in Tregs (Fig. 5G and 5H). These results indicate that SCM-198 might suppresses the EMS progress by downregulating peritoneal Tregs.

### SCM-198 restrains the expansion and function of Tregs induced by estrogen in EMS mice

**Figure 6:**
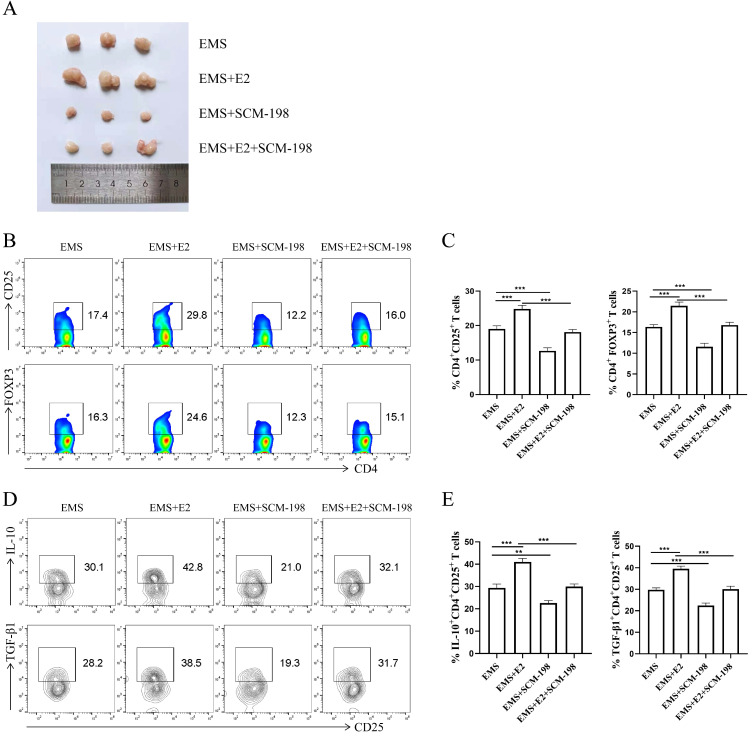
** SCM-198 restrained the expansion and function of Tregs induced by estrogen in EMS mice. (A)** Representative picture of ectopic lesions from EMS mice treated with or without E2 (150 µg/kg), SCM-198 (15 mg/kg) or E2+SCM-198 (150 µg/kg, 15 mg/kg) once a day for one week. **(B, C)** Representative (B) and quantitative (C) flow cytometry results for CD25 and FOXP3 expression in CD4+T cells from peritoneal fluid of EMS mice (n=10). **(D, E)** Representative (D) and quantitative (E) results showing the analysis of IL-10 and TGF-β1 expression in CD4+ CD25+ Tregs from EMS mice (n=10). Data are presented as the mean ± SEM. **P < 0.01, ***P < 0.001.

To further verify SCM-198 alleviated the development of EMS by inhibiting Tregs accumulation, we administrated EMS mice with E2. As shown in Fig. 6A, E2 aggravated the development of EMS, which could be attenuated by SCM-198. In addition, the effects of E2 on Tregs could be reversed by SCM-198 (Fig. 6B-6E).

These data suggest that estrogen contributes to the development of EMS and is favorable for peritoneal Tregs accumulation. SCM-198 administration can abate the progress of ectopic lesions induced by estrogen. Moreover, the influence of estrogen on Tregs expansion and cytokine production (IL-10 and TGF-β1) can also be ameliorated by SCM-198.

## Discussion

The occurrence and development of EMS are closely related to high estrogen and immune disorders in peritoneal environment 25, 26. Assorted immune cells, including NK cells, macrophages and T cells are reported to be dysregulated in patients with EMS. Impaired cytotoxicity of NK cells and reduced phagocytic capacity of macrophages permitted the ectopic endometrium to escape immune surveillance and clearance 10-12, 27.

Tregs, one subpopulation of T cells, has been widely studied in oncology for its immunosuppressive function 28, 29. The researchers also focused on Tregs in EMS for its similar characteristics of benign tumors, such as increased invasion and viability. Several experiments have shown that Tregs are more abundant in peritoneal fluid of EMS patients 17, 30, 31. Besides, accumulated Tregs induced a type 2 immune microenvironment, which accelerated the progress and fibrosis of ectopic lesions 16.

The crosstalk between endocrine and immune systems can affect both physiological and pathological processes 32-34. Plenty of studies have revealed that estrogen possesses immunomodulatory functions in Tregs 35-37. Impaired estrogen signaling impeded the immunosuppressive function of Tregs, which further aggravated inflammatory bowel disease (IBD) 36. Estrogen signaling suppressed bone resorption by facilitating the production of IL-10 and TGF-β1 in Tregs 37. Studies have uncovered that increased concentration of estrogen and upregulated accumulation of Tregs both aggravated the development of EMS. However, it has not been explored whether the expansion of Tregs are resulted from local high estrogen. Exploring the regulation of estrogen signaling on Tregs is important to clarify the pathogenesis of EMS. In the present study, we found Tregs accumulated and estrogen increased in peritoneal fluid of EMS patients. The higher mRNA expression of ERα in Tregs was observed. To investigate whether the enhanced estrogen signaling leaded to the accumulation of Tregs, *in vitro* Tregs differentiation assays were conducted under the treatment of E2 or PPT. The results revealed that activated estrogen signaling upregulated Tregs in EMS.

Except for clarifying the pathogenesis, its urgent to find effective drugs to treat EMS for its high prevalence and recurrence 3-5. SCM-198 is the chemically synthesized leonurine, exerts beneficial roles in various clinical diseases, such as cardio-cerebrovascular diseases and hypercholesterolemia disorders 38, 39. Our previous studies have demonstrated that SCM-198 has protective effects on endometrium oxidative stress and inflammation 21, 22.

Here, we explored the therapeutic effects and the related mechanisms of SCM-198 on EMS. The results showed that SCM-198 obviously downregulated the expansion of Tregs and notably inhibited the activation of estrogen signaling, including decreasing estrogen production in eESCs and suppressing the mRNA level of ERα in Tregs. *In vitro* Tregs differentiation assays revealed that SCM-198 abated the differentiation and cytokine production of Tregs by inhibiting estrogen-ERα signaling. The abnormal conversation between Tregs and eESCs facilitated the invasion and viability of eESCs, which allows EMS to occur and develop rapidly 18, 40. We demonstrated that SCM-198 ameliorated the invasion and decreased the viability of eESCs enhanced by Tregs.

Furthermore, the therapeutic effects of SCM-198 on EMS mice were analyzed. SCM-198 obviously suppressed the growth of ectopic lesions. In consistent with* in vitro* experiments, SCM-198 dramatically downregulated the expansion of Tregs and cytokine production in Tregs (IL-10 and TGF-β1). The therapeutic effects of SCM-198 are based on the repair of endocrine-immune regulation.

It has been reported that estrogen-estrogen receptor (ER) signaling contributed to the survival and apoptotic resistance of eESCs 41-43. Whether the direct inhibitory effect of SCM-198 on the viability of eESCs was achieved by estrogen-ER signaling needs further research. In addition, estrogen itself could also discipline the immune system in EMS. For example, estrogen impaired phagocytic function of macrophages 10 and reduced the cytotoxicity of NK cells 43. Whether SCM-198 could relieve the inhibitory effects of estrogen on the immune clearance function of macrophages, NK cells and other immune cells is worthy of further study.

**Figure 7:**
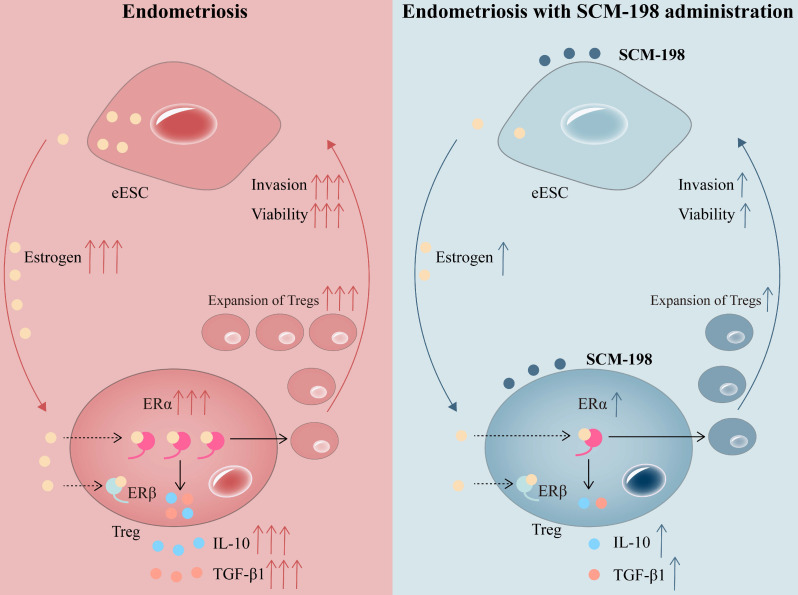
** Schematic diagram showing the therapeutic mechanism of SCM-198 on EMS.** In the peritoneal fluid of EMS patients, higher concentration of estrogen and accumulated Tregs are presented. Activated estrogen-ERα signaling potentiates the expansion of Tregs and their cytokine production (IL-10 and TGF-β1). In turn, cumulative Tregs facilitated the invasion and viability of eESCs. SCM-198 reduces the accumulation of Tregs by inhibiting estrogen-ERα signaling activation through inhibiting estrogen secretion of eESCs and suppressing ERα expression of Tregs. What's more, SCM-198 restrains the invasion and viability of eESCs reinforced by the accumulated Tregs.

In conclusion, our research shows that higher concentration of estrogen and accumulated Tregs are presented in the peritoneal fluid of EMS patients. The overactivation of estrogen-ERα signaling potentiates the expansion of Tregs. SCM-198 abates the accumulation of Tregs by inhibiting estrogen-ERα signaling activation through decreasing estrogen secretion of eESCs and downregulating ERα expression of Tregs. What's more, SCM-198 restrains the growth of ectopic lesions and downregulates the accumulation of Tregs in EMS mice (Fig. 7). To sum up, this study provides a theoretical basis for SCM-198 on the treatment of EMS.

## Supplementary Material

Supplementary figure.

## Figures and Tables

**Table 1:** Characteristics of recruited participants

Subjects	Non-EMS	EMS	*p*
Number	30	46	-
Age range (years)	23-46	22-45	-
Age mean^a^	35.6±0.99	34.98±0.87	ns
Cyst diameter size (cm)^b^	-	5.79±0.91	-
**rAFS stage (n(%))**			
I	NA	0	-
II	NA	0	-
III	NA	18(39.1%)	-
IV	NA	28(60.9%)	-
**Menstrual cycle (n (%))**			
Proliferative phase	12(40%)	16(34.78%)	-
Secretory phase	18(60%)	30(65.22%)	-
Treatment history	-	-	-

^a^Mean ± standard error of the mean (SEM); ^b^Mean ± standard deviation (SD); rAFS: revised American Fertility Society.

## Abbreviations

EMS: endometriosis
Tregs: regulatory T cells
ESCs: endometrial stromal cells
ERα: estrogen receptor α
PCOS: polycystic ovary syndrome
ePF: EMS peritoneal fluid
nPF: non-EMS peritoneal fluid
FBS: fetal bovine serum
eESCs: endometrial stromal cells
cDNA: complementary DNA
PBMCs: peripheral blood mononuclear cells
E2: 17β-estradiol
PPT: propyl pyrazole triol
RIPA: radio-immunoprecipitation assay
IBD: inflammatory bowel disease
ERβ: estrogen receptor β
ER: estrogen receptor
