# Supplementary figures and images for: SCM-198 Alleviates Endometriosis by Suppressing Estrogen-ERα mediated Differentiation and Function of CD4+CD25+ Regulatory T Cells
Source: Int J Biol Sci. 2022 Feb 21;18(5):1961–73. doi: 10.7150/ijbs.68224 (PMC8935231; doi:10.7150/ijbs.68224)

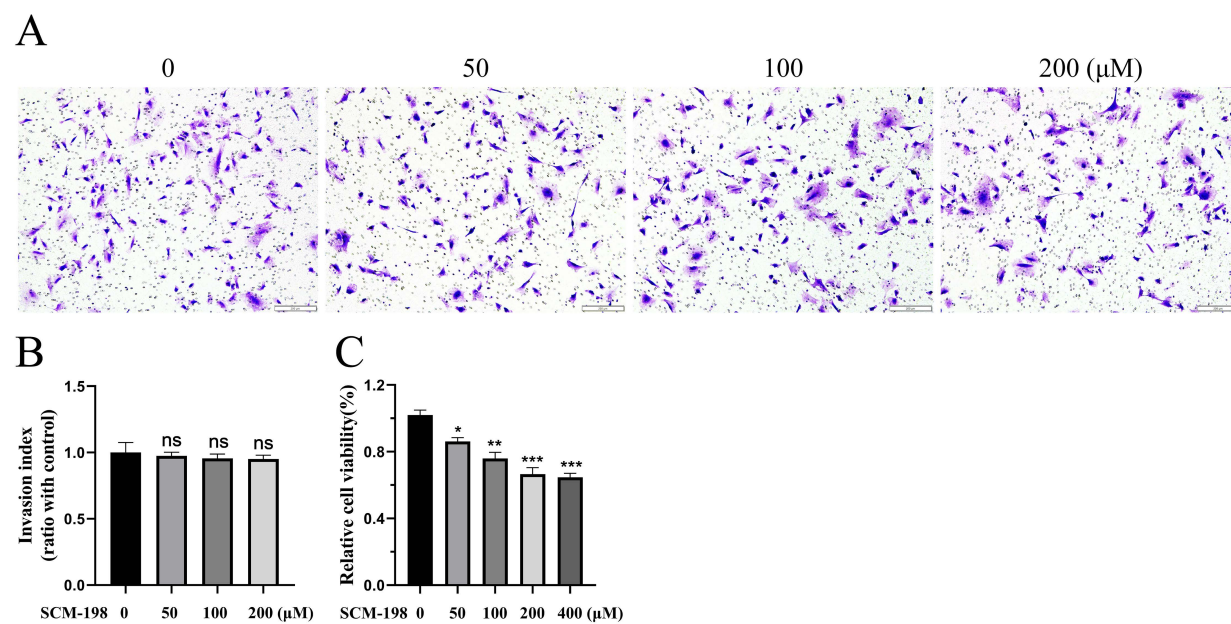

Figure S1. The direct effects of SCM-198 on the invasion and viability of eESCs

Supplement: Supplementary file 1 — Supplementary figure. [file ijbsv18p1961s1.pdf]
